# Fecal microbiota transplantation alleviates radiation enteritis by modulating gut microbiota and metabolite profiles

**DOI:** 10.17305/bb.2025.11835

**Published:** 2025-02-03

**Authors:** Qin Ding, Jing Xue, Nan Li, Hu Zhihui, Song Jianbo

**Affiliations:** 1 Shanxi Bethune Hospital, Shanxi Academy of Medical Sciences, Third Hospital of Shanxi Medical University, Tongji Shanxi Hospital, Taiyuan, China; 2 Third Hospital of Shanxi Medical University, Shanxi Bethune Hospital, Shanxi Academy of Medical Sciences, Tongji Shanxi Hospital, Taiyuan, China; 3 Shanxi Provincial Key Laboratory for Translational Nuclear Medicine and Precision Protection, Taiyuan, China

**Keywords:** Fecal microbiota transplantation, FMT, radiation enteritis, RE, gut microbiota, metabolite profiles, mechanisms

## Abstract

This study investigates the safety and underlying mechanisms of fecal microbiota transplantation (FMT) in treating radiation enteritis (RE). A rat model of RE was established with six groups: NC, RT, H-FMT, modified FMT (M-FMT), L-FMT, and BTAC. The therapeutic effects of FMT were assessed using the Disease Activity Index (DAI), histological analysis, and biochemical tests, including ink-propelling, xylitol exclusion, and enzyme-linked immunosorbent assay (ELISA). Gut microbiota alterations and fecal metabolism were analyzed via 16S rDNA sequencing and targeted metabolomics. The results demonstrated that FMT, particularly in the M-FMT group, effectively alleviated RE by reducing DAI scores, histological damage, and inflammatory markers while enhancing enzyme activity, superoxide dismutase (SOD) levels, and intestinal absorption. FMT also modulated gut microbiota composition, increasing beneficial species, such as *Blautia wexlerae* and *Romboutsia timonensis* while decreasing *Enterococcus ratti*. Metabolomics analysis revealed that FMT influenced niacin, nicotinamide, and starch metabolism, with notable changes in pantothenic acid and fatty acid levels. Spearman correlation analysis further indicated that these microbial shifts were associated with improved metabolic profiles. Overall, FMT mitigates RE by regulating gut microbiota and metabolites, with pantothenic acid and fatty acids emerging as potential therapeutic targets. Further research is needed to explore the underlying mechanisms in greater detail.

## Introduction

Radiotherapy is a cornerstone in managing abdominal and pelvic malignancies [1, 2]. Despite continuous advancements in radiotherapy technology and precision, unintended effects on normal intestinal tissues persist. Radiation enteritis (RE) is a common complication in patients receiving pelvic radiation therapy, particularly in prostate, rectal, and gynecological cancer treatments. Acute RE (ARE) occurs in approximately 20%–30% of patients after radiotherapy, whereas chronic RE (CRE) may manifest months or even years later, with a prevalence of about 5%–15% [3, 4]. Clinical manifestations of RE range from mild to severe and include rectal bleeding, diarrhea, urgency, anal pain, and mucus discharge. In severe cases, long-term complications, such as intestinal stenosis and fibrosis can significantly impact a patient’s quality of life. Treatment primarily focuses on symptom relief and preventing further rectal tissue damage. Conservative approaches include dietary modifications, anti-inflammatory medications, and rectal suppositories or enemas [5, 6]. Antibiotics, by controlling bacterial overgrowth and modulating the intestinal microbiota, have been shown to alleviate RE symptoms [7]. However, prolonged antibiotic use may disrupt microbial diversity, increasing the risk of dysbiosis and long-term gastrointestinal (GI) complications. For severe or refractory cases, additional interventions, such as hyperbaric oxygen therapy, surgical management, or argon plasma coagulation may be necessary. Emerging therapies, such as stem cell transplantation, have shown promise in reducing inflammation and promoting tissue repair. Notably, the first clinical study of a stem cell injection (TH-SC01) for RE began in June 2023 [8]. Despite these treatment options, managing RE remains challenging, with up to 90% of patients undergoing pelvic radiotherapy experiencing permanent bowel habit changes that significantly reduce their quality of life. Moreover, RE can lead to interruptions in oncologic treatment, negatively impacting cancer outcomes and prognosis [9, 10]. Therefore, a deeper understanding of RE and the development of more effective therapeutic strategies are crucial to improving patient outcomes. Recent studies highlight the key role of gut microbiota in maintaining intestinal homeostasis [11, 12]. Ionizing radiation has been shown to reduce gut microbiota diversity and alter microbial composition, most consistently increasing the relative abundance of Proteobacteria [13, 14]. These changes disrupt intestinal microecology, trigger inflammatory responses, and contribute to intestinal damage. Such findings suggest that gut microbiota may play a central role in RE pathogenesis, offering potential therapeutic targets [15, 16]. Fecal microbiota transplantation (FMT) is an emerging approach that aims to restore gut microbiota by transferring intestinal flora from healthy donors to patients [17, 18]. First applied by Eiseman et al. [19] to treat pseudomembranous enteritis caused by antibiotics, FMT demonstrated significant clinical benefits. More recently, Ding et al. [20] reported that FMT safely and effectively alleviated intestinal symptoms and mucosal injury in a small cohort of five RE patients. Additionally, 16S rRNA sequencing by Cui et al. [21] revealed that FMT increased the relative abundance of beneficial microbial populations, including Bacteroidetes and Lactobacillus, while enhancing Prevotella levels at the genus level. However, the precise mechanisms by which FMT modulates the gut microbiome in RE remain largely unexplored.

The GI tract contains numerous metabolites essential for communication between the host and gut microbiota. These metabolites include bioactive molecules—such as short- and long-chain fatty acids, indole derivatives, and polysaccharide A—that are produced or modified endogenously by the intestinal flora. These molecules play a critical role in maintaining intestinal barrier integrity and immune system homeostasis [22–24]. Numerous studies have shown that short-chain fatty acids (SCFAs), especially butyrate, enhance the function of immune cells such as Tregs in the gut, thereby alleviating colitis. Butyrate is even used in enema treatments for inflammatory bowel disease to suppress excessive immune responses [25]. Valeric acid (VA), another SCFA, is produced by specific gut bacteria during the fermentation of fiber-rich foods. Li et al. [26] demonstrated that VA reduces inflammation and protects the gut microbiota, exerting a protective effect against radiation-induced hematopoietic and intestinal damage. Additionally, Zhao et al. [27] reported that intestinal dysbiosis exacerbates psoriasis by altering fatty acid metabolism. Vitamin D also plays a key role in gut health by increasing microbial diversity and promoting the growth of probiotics, such as Akkermansia and Bifidobacteria, which help maintain immune homeostasis [28]. Based on these findings, we hypothesized that FMT may modulate metabolic functions by inducing changes in gut microbiota composition, ultimately contributing to the alleviation of RE symptoms. In this study, we first determined the optimal dose for constructing the RE model and then used this model to investigate the potential of FMT as a treatment. To gain a comprehensive understanding of microbiota dynamics during FMT, we employed a combined approach involving non-targeted metabolomics. The design of this study aims to provide a theoretical foundation for the clinical application of FMT in treating RE, offering scientific guidance for improved patient care and ultimately enhancing the rehabilitation and quality of life of individuals undergoing abdominal and pelvic tumor radiotherapy.

## Materials and methods

### Animals and irradiation procedure

Sprague–Dawley (SD) male rats (6–8 weeks old, 200 ± 2 g) were obtained from Beijing Viton Lihua Laboratory Animal Technology Co. Upon arrival, they were housed in a well-ventilated room under specific pathogen-free (SPF) conditions, with unrestricted access to water and SPF-grade pellet feed. The ambient temperature was maintained at 22 ± 2 ^∘^C, relative humidity at 60 ± 5%, and a 12-h light–dark cycle. Except for the no-treatment group (NTG), all rats were anesthetized via intraperitoneal injection of chloral hydrate (3.5 mL/kg) and positioned supine on a board for immobilization. X-ray irradiation was performed using a British Elekta Synergy linear accelerator (6 MeV), targeting the area from the midline to the pubic symphysis. The irradiation field was set at 3 cm × 4 cm, with the remaining areas shielded by a 5-cm thick lead plate. The dose rate was 3 Gy/min, with a radiation output of 600 MU/min and a source-to-skin distance of 100 cm.

### Grouping and treatments

Dose conversion and optimization were based on the standard clinical protocol for a 500-mL enema for ulcerative colitis, in which 100 g of feces is dissolved in 500 mL of sterile saline. For an adult weighing 60 kg, the dose is 1.67 g/kg/day. Using the FDA-recommended body surface area conversion factor of 6.25 for humans to rats, the equivalent rat dose was calculated as 10.438 g/kg/day [29–31]. For a 200-g rat, this corresponds to a daily dose of 2.0876 g, with an initial enema volume estimate of 10.438 mL. However, factors, such as intestinal volume, drug loss during administration, potential irritation, and enema solution concentration were considered for further optimization. By adjusting the diluent-to-feces ratio from 1:5 to 1:3.1, the final enema volume was reduced to 6.4 mL per 200 g rat (or 3.2 mL per 100 g rat). Based on this optimization, three dose groups were established: Medium-dose group (MDG): 10.438 g/kg/day; Low-dose group (LDG): 5.219 g/kg/day (half of MDG); High-dose group (HDG): 20.876 g/kg/day (twice MDG). Additionally, the bifidobacterium triple active capsule group (BTACG) received 0.175 g/kg/day, while the normal treatment group and model group (MOD) were given 0.9% phosphate-buffered saline enemas. All groups followed a twice-daily enema regimen for a total of six treatments.

### Disease activity index (DAI)

Monitor the rats’ diet, water consumption, fur luster, mental status, and perianal cleanliness daily. Weigh each group of rats daily and document fecal characteristics, including the presence of occult or visible blood. DAI was calculated using the following formula: DAI ═ (body weight score + stool consistency score + bleeding score) / 3, as detailed in Table 1.

**Table 1:** DAI scoring scale

**Fraction of body mass loss (%)**	**Stool properties**	**Occult blood/hemorrhage**
0	Well-formed pills	Negative faecal occult blood
1-5	Somewhat soft	Negative/positive fecal occult blood
6-10	Soft	Some amount of fecal occult blood
11-15	Unformed or mild diarrhea	Visible rectal bleeding
>15	Severe watery diarrhea	Severe rectal bleeding

DAI: Disease activity index.

### Ink propelling and D-xylose excretion test

Urine was collected from rats within 5 h before radiation exposure and on the 4th, 5th, 6th, and 14th days post-exposure. Before euthanizing the rats in each group, an ink propulsion test was performed by administering ink via gavage [32]. The ileal mucosal absorption capacity was comprehensively evaluated using the following formulas: Xylose excretion rate ═ (urinary xylose content / administered xylose content) × 100%; Small intestine propulsion rate ═ (distance from the anterior end of the charcoal to the pylorus / total length of the small intestine) × 100%.

### Enzyme-linked immunosorbent assay (ELISA)

Ileal tissues were collected from the rats, and the levels of inflammatory factors (TNF-α, IL-1β, and IL-6) as well as oxidative stress markers (ROS, NO, and SOD) were measured using an ELISA kit from Andy Gene Company. Detailed procedures are available in the kit’s instruction manual.

### Hematoxylin–eosin (HE) staining

Ileal tissues were collected and fixed in 4% paraformaldehyde for 24 h. The tissues were then embedded in paraffin wax and subjected to HE staining.

### 16SrRNA sequencing and bioinformatics analysis

Fresh fecal pellets from both irradiated and control mice were collected, weighed, and stored in cryopreservation tubes at –80 ^∘^C until processing. 16S rRNA sequencing was performed by Shanghai Personal Biotechnology Co., Ltd. (Shanghai, China) using full-length triple sequencing, with a 100% sequence similarity threshold to define amplicon sequence variants (ASVs). Species annotation of ASVs was based on clustering results. To compare the composition, abundance, and diversity of intestinal flora among groups, both alpha and beta diversity analyses were conducted. Hallmark genera for each group were identified using linear discriminant analysis effect size (LEfSe). Functional pathway analysis, based on the Kyoto Encyclopedia of Genes and Genomes (KEGG) database, assessed the average abundance of all samples within secondary functional pathways.

### High-performance liquid chromatography-tandem mass spectrometry (UHPLC-MS/MS)

Fecal samples were precooled, vortexed, and centrifuged before the supernatant was extracted and loaded into liquid-phase vials. The UHPLC-MS/MS technique was employed for analysis at the Large Instrument Center of Shanxi University, following the parameters specified in the relevant manual. Raw data were preprocessed, with metabolite levels normalized and interpolated, while features with relative standard deviations greater than 30% were excluded. Mass spectrometry data were identified using the Metlin database, retaining only metabolites with MS/MS fragment scores above 30. The corrected data were then subjected to multivariate statistical analyses, including principal component analysis (PCA). Differential metabolites were identified based on the criteria of variable importance in projection (VIP) > 1, *P* < 0.05, and fold change (FC) ≥ 1.2 or ≤ 0.83. Pathway enrichment analyses were performed using metabolomic pathway analysis (MetPA).

### Ethical statement

This study was approved by the Regional Ethics Committee for Animal Experiments at Shanxi Bethune Hospital (Grant number: 202103021224365).

### Quantification and statistical analysis

Statistical package for the social sciences (SPSS) software was used for statistical analysis, and the measurement data were expressed as x ± s. Differences between groups were analyzed by one-way analysis of variance (ANOVA), and a *t*-test was used to determine the differences between the two groups. The correlation coefficient between microbiomics and metabolomics data was calculated using Spearman correlation analysis, and *P* < 0.05 was considered statistically significant.

## Results

### 12 Gy is most appropriate for RE modeling

**Figure 1.**
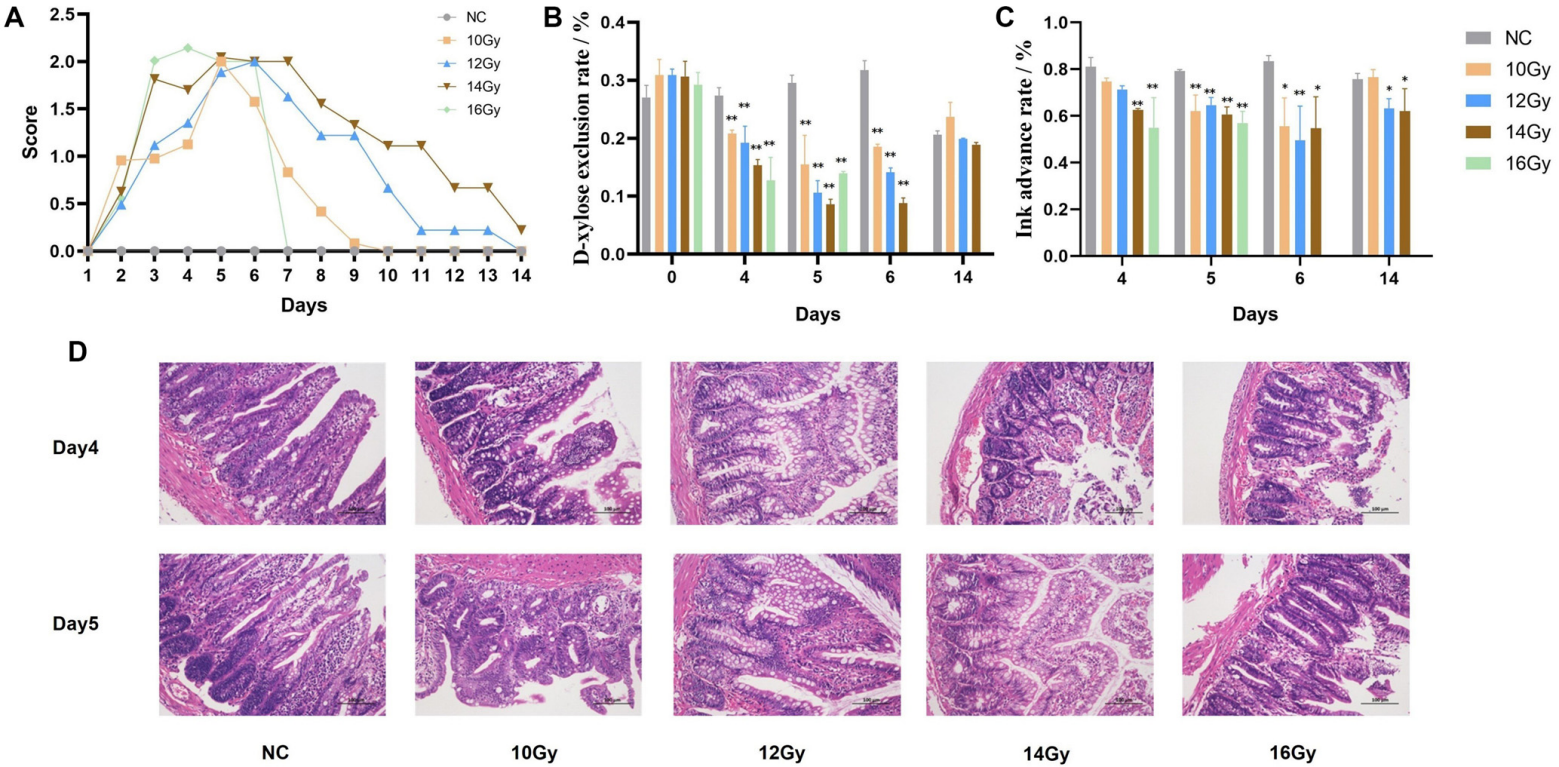
**Dose exploration for constructing the RE model: NC, 10 Gy, 12 Gy, 14 Gy and 16 Gy.** (A) DAI scores; (B) D-xylose excretion test; (C) Ink-propelling test; (D) HE staining of ileal (100 um). ^*^, compared with the NC, *P* < 0.05; ^**^, compared with the NC, *P* < 0.01. RE: Radiation enteritis; DAI: Disease activity index; HE: Hematoxylin–eosin.

To determine the optimal radiation dose for establishing an RE model, SPF-grade male rats were selected and randomly divided into five groups: NC (10), 10 Gy (17), 12 Gy (17), 14 Gy (17), and 16 Gy (17). Compared with the NC group, the DAI score of the RT group was significantly increased and showed a clear dose-dependent relationship. The general condition of the rats began to improve by day 7 post-irradiation (Figure 1A). Irradiation led to a decrease in the absorption capacity of the small intestinal mucosa, particularly in the 12 and 14 Gy groups (*P* < 0.01, Figure 1B). Similarly, small intestinal propulsion rates continued to decline after irradiation, reaching their lowest point by day 6. However, by day 14 post-irradiation, some recovery of intestinal mucosal damage was observed (Figure 1C). HE staining of ileal tissue post-irradiation revealed varying degrees of intestinal damage across all dose groups, including severe destruction of intestinal villi, epithelial damage, noticeable congestion and edema of the intestinal mucosa, and significant inflammatory cell infiltration. The 16 Gy group exhibited the most severe damage (Figure 1D). Mortality rates increased with radiation dose: no deaths occurred in the 10 Gy group, while there were 3 deaths in the 12 Gy group, 5 in the 14 Gy group, and 8 in the 16 Gy group. Based on symptom severity and mortality rates, we conclude that 12 Gy is the most appropriate dose for RE modeling.

### FMT could effectively ameliorate radiation-induced intestinal injury

**Figure 2.**
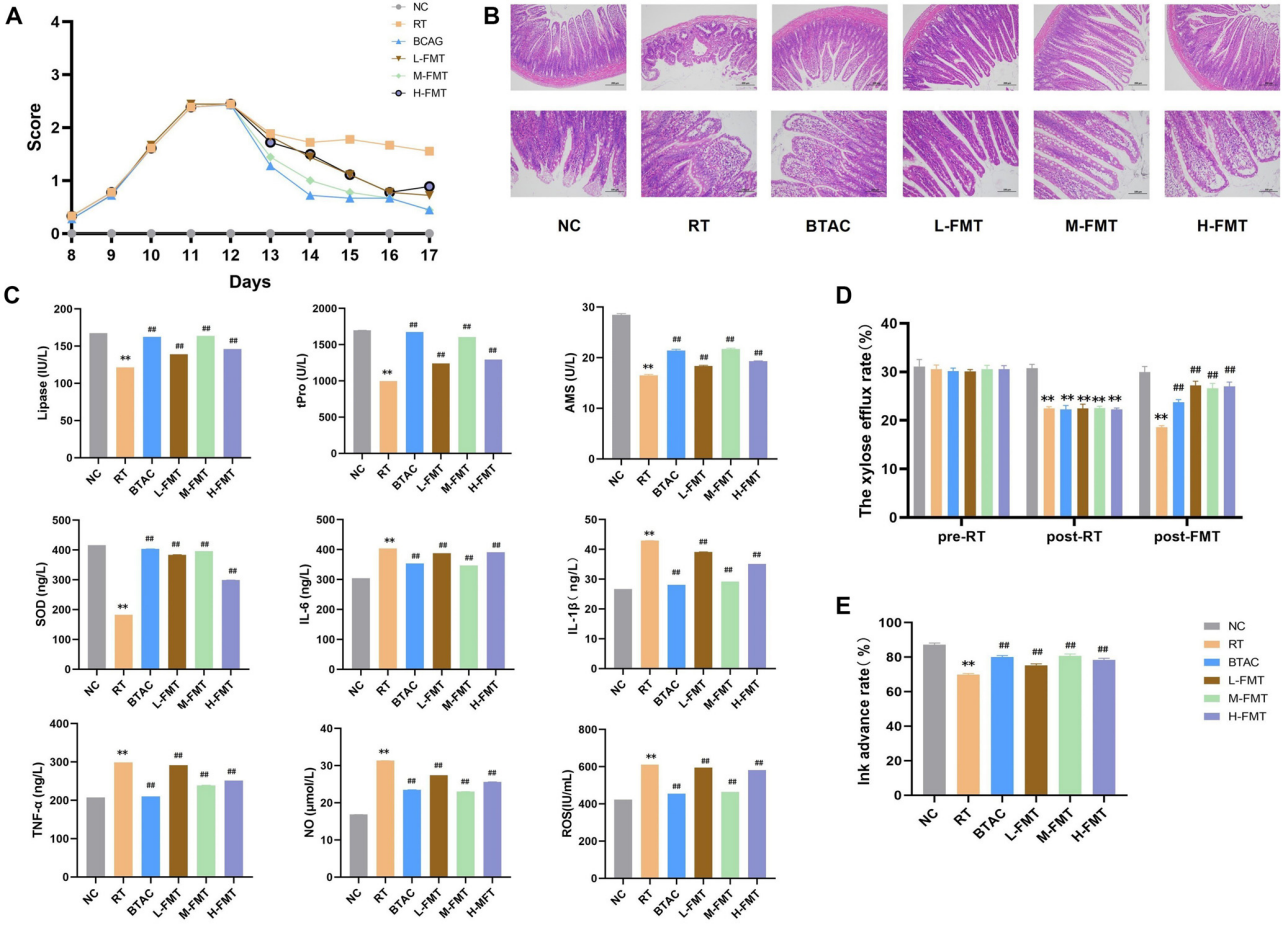
**Efficacy of FMT in RE: NC, RT, BTAC, L-FMT, M-FMT, and H-FMT.** (A) DAI scores; (B) HE staining of ileal tissue; (C) The level of lipase, AMS and t-Pro, TNF-α, IL-1β, IL-6, ROS, and NO; (D) D-xylose excretion test; (E) Ink-propelling test. ^*^, compared with the NC, *P* < 0.05; ^**^, compared with the NC, *P* < 0.01; ^#^, compared with the RT, *P* < 0.05; ^##^, compared with the RT, *P* < 0.01. FMT: Fecal microbiota transplantation; RE: Radiation enteritis; DAI: Disease activity index; HE: Hematoxylin–eosin; M-FMT: Modified FMT.

To evaluate the efficacy of FMT in RE, SPF-grade male rats were selected and randomly assigned to six groups: NC, RT, H-FMT, modified FMT (M-FMT), L-FMT, and BTAC. All groups, except NC, underwent irradiation at a dose of 12 Gy. Compared to the NC group, the RT group exhibited a significant increase in DAI scores. However, the intervention groups (H-FMT, M-FMT, L-FMT, and BTAC) showed significantly lower DAI scores than the RT group (*P* < 0.01, Figure 2A). HE staining results revealed that the intestinal mucosa in the NC group remained intact, with thick, well-structured intestinal villi and no evident inflammatory cell infiltration. In contrast, the RT group displayed a thinned intestinal wall, disorganized and underdeveloped villi, and clear inflammatory cell infiltration, particularly neutrophils. Compared to the RT group, the L-FMT group exhibited more neatly arranged intestinal villi, while the M-FMT group showed increased villus length and a more organized structure. The H-FMT group demonstrated an increase in goblet cells and glandular structures, which were also neatly arranged. Similarly, the BTAC group had an intact intestinal mucosal epithelium, abundant glands, and no apparent inflammatory cell infiltration, indicating partial restoration of intestinal integrity in all intervention groups (Figure 2B). Biochemical analysis showed that, compared to the NC group, the RT group had significantly lower levels of digestive enzymes (lipase, AMS, and t-Pro) and SOD (*P* < 0.01), along with significantly elevated levels of inflammatory markers, including TNF-α, IL-1β, IL-6, ROS, and NO (*P* < 0.01). In contrast, the intervention groups exhibited decreased inflammatory marker levels and restored digestive enzyme and SOD levels compared to the RT group (*P* < 0.01, Figure 2C). The xylose efflux rate and small intestinal propulsion rate were also assessed at different time points. Before irradiation, no significant differences were observed among the groups. After irradiation, the xylose efflux rate significantly decreased compared to the NC group (*P* < 0.01), but the administration of FMT and BTAC restored it to near-normal levels (*P* > 0.05, Figure 2D). Similar results were observed for small intestinal propulsion (Figure 2E). Notably, the improvement in RE was comparable between the M-FMT and BTAC groups.

### FMT enhances the diversity of rat intestinal flora

**Figure 3.**
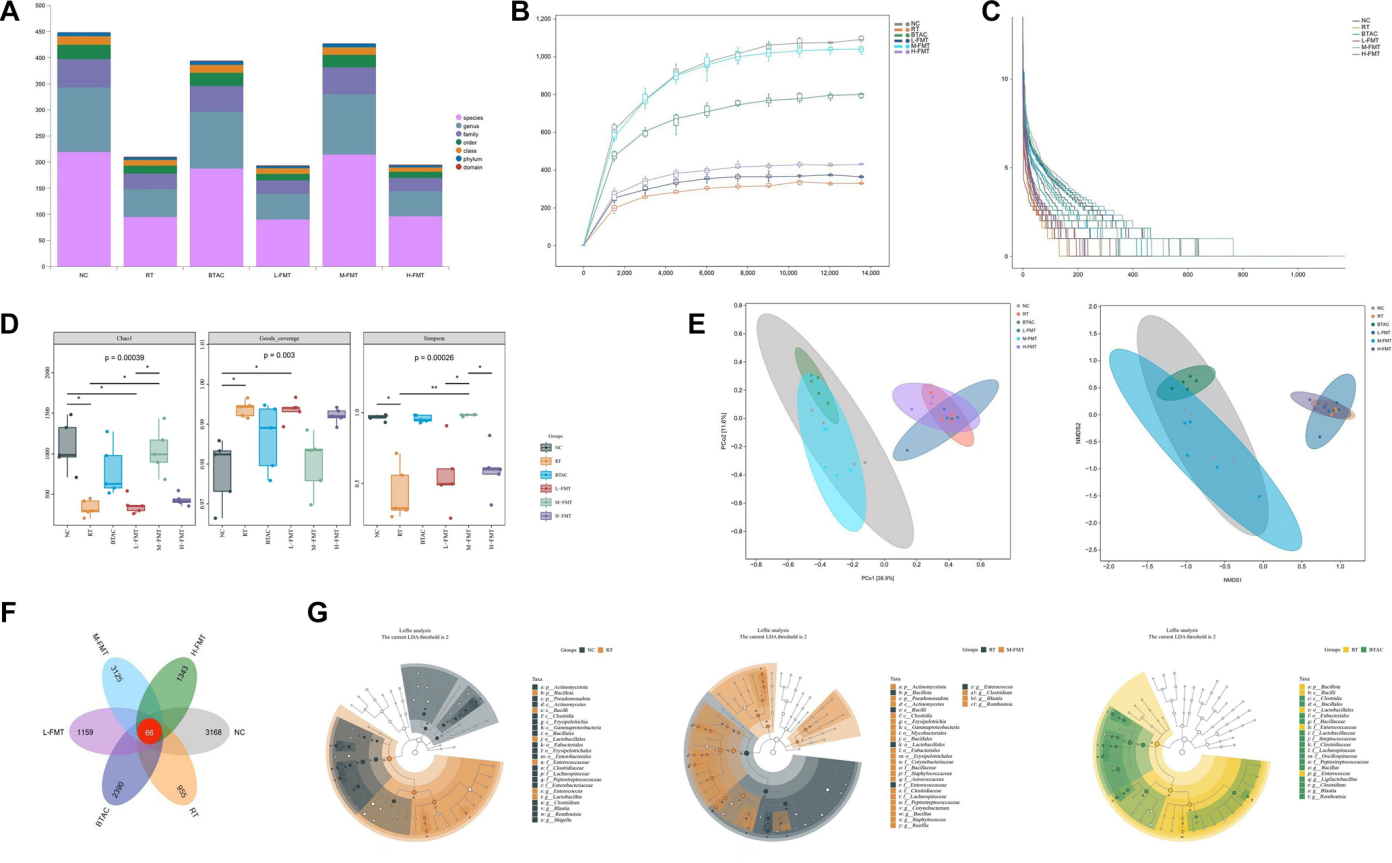
**Overall gut microbiota analysis.** (A) Taxonomic annotation; (B) The rarefaction curves; (C) The rank abundance curve; (D) The Chao, Shannon, and Simpson index; (E) PCoA and NMDS index; (F) Petal plot; (G) LEfSe analysis between NC, RT, BTAC, and M-FMT. ^*^, compared with the NC, *P* < 0.05; ^**^, compared with the NC, *P* < 0.01. M-FMT: Modified fecal microbiota transplantation; LEfSe: Linear discriminant analysis effect size; PCoA: Principal coordinate analysis; NMDS: Nonmetric multidimensional scaling.

With the positive effect of FMT on RE established, we performed 16S rRNA sequencing of fecal samples to explore the potential mechanisms underlying this improvement. A total of 1,514,416 valid sequences were obtained, with an average sequence length of 2 bp. Taxonomic annotation revealed significant differences in microbiota composition among groups (Figure 3A). The rarefaction curve plateaued as the number of sequence reads increased, indicating sufficient sequencing depth to capture microbial diversity and composition (Figure 3B). This trend confirmed that additional reads would have minimal impact on community structure, demonstrating the reliability and representativeness of the data. Rank abundance curves exhibited a smooth downward trend, suggesting evenly distributed microbial diversity without dominance by a single population (Figure 3C). Community diversity was further assessed through α-diversity analysis. The Chao index, which estimates community richness, significantly decreased in the RT group but was restored in the BTAC and M-FMT groups. The Shannon and Simpson indices, which reflect gut microbiota diversity, were significantly reduced in RT compared to NC but showed recovery in BTAC and M-FMT (Figure 3D). β-diversity analysis, conducted via principal coordinate analysis (PCoA) and nonmetric multidimensional scaling (NMDS), illustrated similarities and differences in community composition (Figure 3E). Closer clustering indicated greater similarity, and results showed clear separation among the six groups. Similarity analysis further confirmed that within-group differences were smaller than between-group differences (*R* ═ 0.11823, *P* ═ 0.001).

To assess species distribution across different groups, a petal plot was generated, revealing that the highest number of ASVs/operational taxonomic units (OTUs) was observed in the NC group (3,168), followed by the M-FMT (3,125), BTAC (2,390), H-FMT (1,343), L-FMT (1,159), and RT (955). A total of 66 ASVs/OTUs were shared among all six groups (Figure 3F). Additionally, LEfSe analysis was conducted to identify differentially abundant fecal bacterial taxa among the groups. Compared to NC, the RT model exhibited fewer differential marker bacteria, whereas the BTAC and FMT groups showed a higher number. This suggests that FMT may influence the diversity of the rat intestinal flora, potentially contributing to its effects (Figure 3G).

### FMT improves gut microbial imbalance in rats

**Figure 4.**
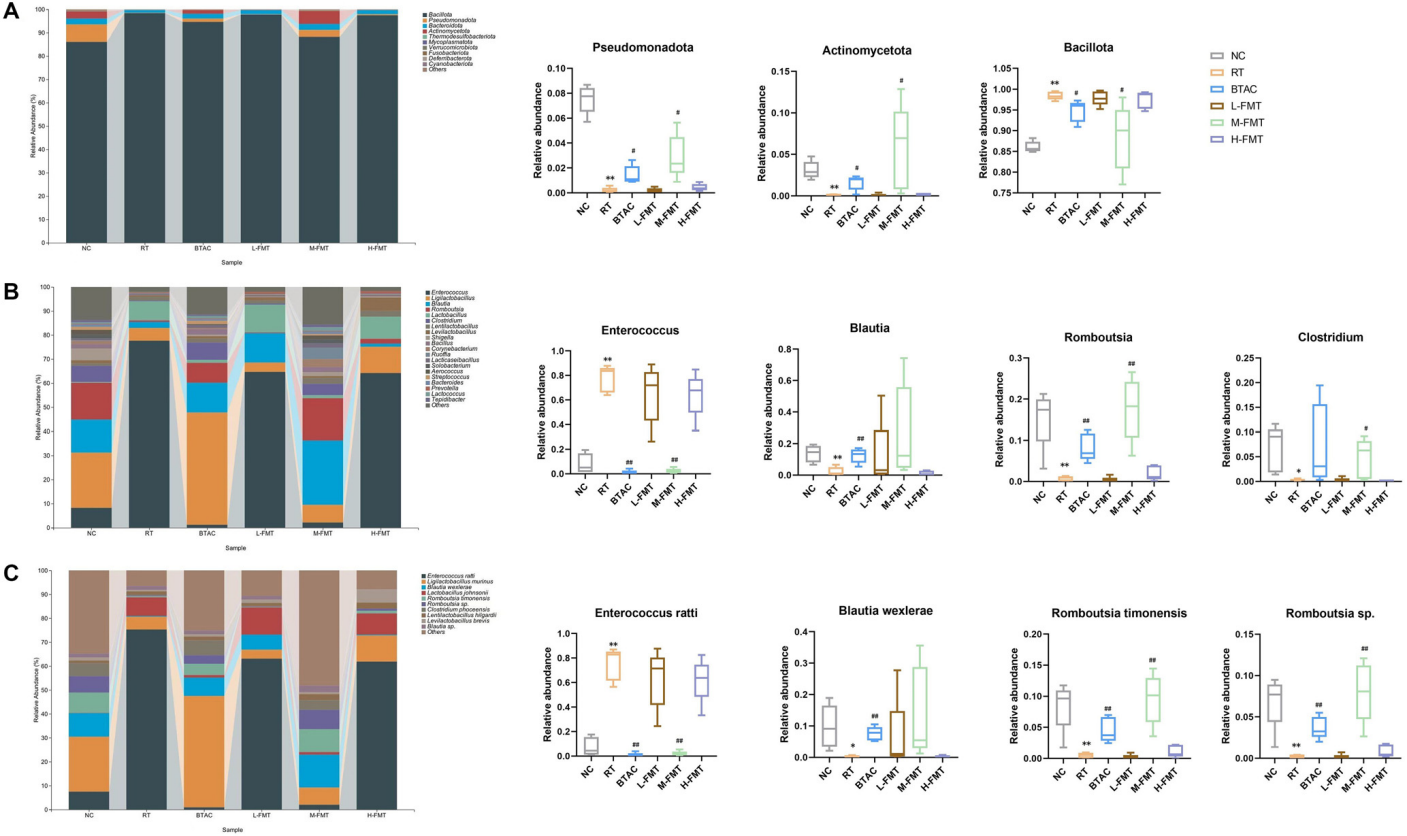
**Taxonomy analysis of gut microbiota.** (A) Phylum; (B) Genus; (C) Species. ^*^, compared with the NC, *P* < 0.05; ^**^, compared with the NC, *P* < 0.01; ^#^, compared with the RT, *P* < 0.05; ^##^, compared with the RT, *P* < 0.01.

Since M-FMT and BTAC were more advantageous than other dose groups and more similar to NC, we focused on the differences among NC, RT, BTAC, and M-FMT. Species annotation of all sequences indicated that the number of ASVs/OTUs was significantly reduced in RT. In contrast, BTAC and M-FMT restored the number of ASVs/OTUs in rats with RE (Figure 4). At the phylum level (Figure 4A), compared to NC, *Bacillota* was significantly increased in RT (*P* < 0.01), while *Pseudomonadota* and *Actinomycetota* were significantly decreased (*P* < 0.01). BTAC and M-FMT exhibited the opposite trend, with a decrease in *Bacillota* and an increase in *Pseudomonadota* and *Actinomycetota* (*P*< 0.05). At the genus level (Figure 4B), NC was dominated by *Romboutsia*, *Blautia, Enterococcus*, and *Clostridium* (approximately 21%, 19%, 13%, and 11%, respectively). Compared to NC, Enterococcus was significantly increased in RT (*P* < 0.01), while *Blautia*, *Romboutsia*, and *Clostridium* were significantly decreased (*P* < 0.05). Compared to RT, *Enterococcu*s was significantly decreased in BTAC and M-FMT (*P* < 0.01), whereas *Romboutsia* was significantly increased (*P* < 0.01). Blautia was significantly elevated in BTAC (*P* < 0.01), while only a decreasing trend was observed in RT (*P* > 0.05). Unlike *Blautia*, *Clostridium* increased significantly in M-FMT (*P*< 0.05), whereas BTAC showed an increasing trend without statistical significance. At the species level (Figure 4C), NC was dominated by *Blautia wexlerae*, *Enterococcus ratti*, *Romboutsia timonensis*, and *Clostridium phoceensis* (approximately 19%, 13%, 12%, and 11%, respectively). Compared to NC, *Enterococcus ratti* was significantly increased in RT (*P* < 0.01), while *Blautia wexlerae*, *Romboutsia timonensis*, and *Romboutsia sp.* were significantly decreased (*P* < 0.05). In BTAC and M-FMT, *Enterococcus ratti* was significantly decreased (*P* < 0.01), while *Romboutsia* timonensis and *Romboutsia sp*. were significantly increased (*P* < 0.01). These results suggest that FMT-induced changes in the microbiota may be one of the mechanisms underlying FMT’s effectiveness in treating RE.

### FMT altered the fecal metabolic profile of RE rats

**Figure 5.**
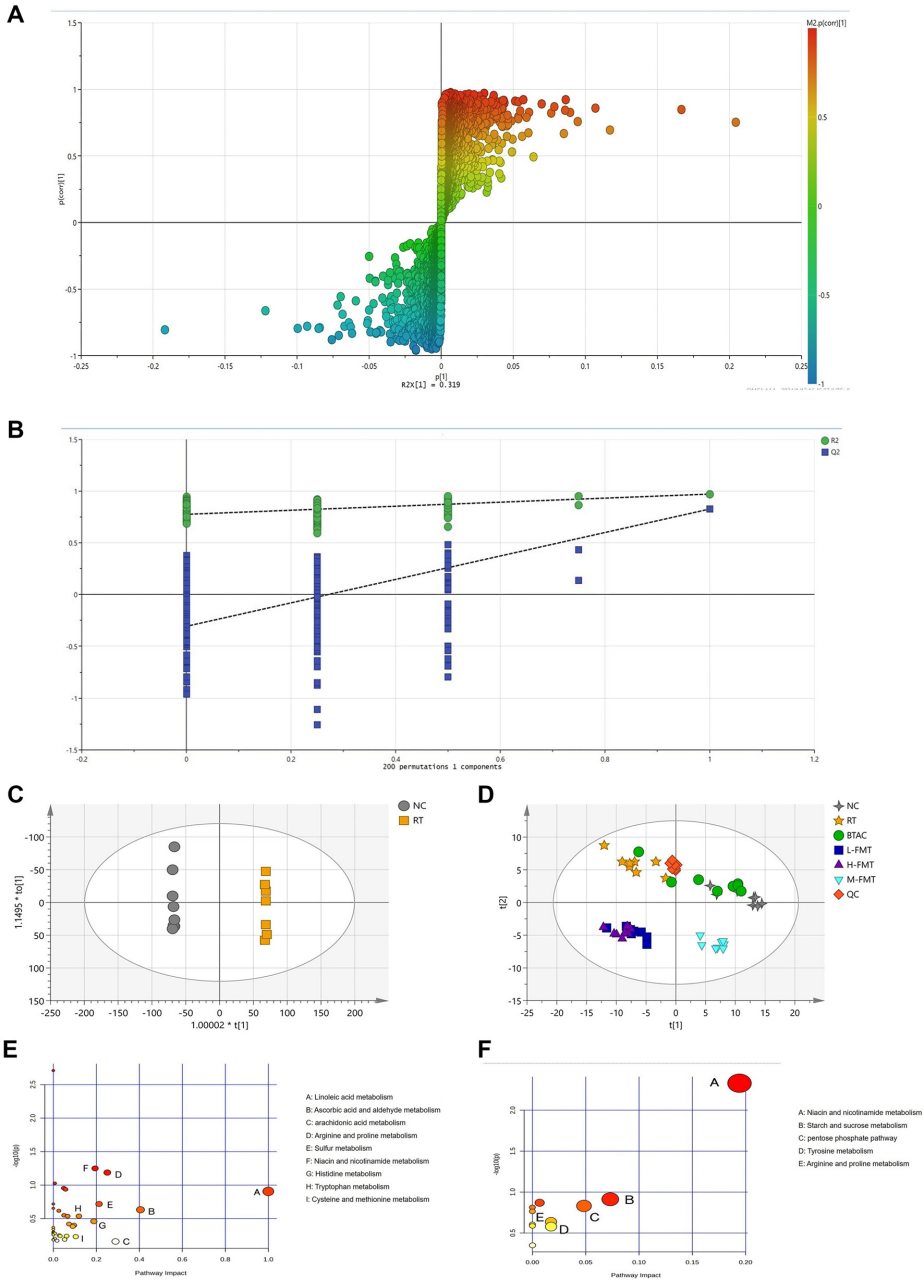
**Metabolic analysis of fecal samples.** (A) Load diagram; (B) The model parameters of permutation analysis; (C and D) OPLS-DA scatter plot between groups; (E) KEGG analysis of differential metabolites between NC and RT; (F) KEGG analysis metabolites callback by the M-FMT. ^*^, compared with the NC, *P* < 0.05; ^**^, compared with the NC, *P* < 0.01; ^#^, compared with the RT, *P* < 0.05; ^##^, compared with the RT, *P* < 0.01. M-FMT: Modified fecal microbiota transplantation; KEGG: Kyoto Encyclopedia of Genes and Genomes.

In the above analysis, we focused on the diversity and species composition of the microbiota. To explore its potential functions, we performed a Phylogenetic Investigation of Communities by Reconstruction of Unobserved States (PICRUSt2) analysis. KEGG analysis indicated that the microbiota primarily influenced metabolic pathways, including carbohydrate metabolism, cofactor and vitamin metabolism, and amino acid metabolism. To further investigate changes in metabolites, we analyzed fecal samples using liquid chromatography-mass spectrometry (LC-MS)-based untargeted metabolomics. Permutation analysis showed an upward trend in model parameters, suggesting that the models were stable and accurately predictive (Figure 5A and 5B). The OPLS-DA scatter plot revealed clear separation between groups, indicating significant differences in fecal metabolites (Figure 5C and 5D). Next, we removed unannotated and repeated components. Metabolites with an FC ≥1.2 or ≤0.83, a VIP value >1, and a *P* value <0.05 were considered differential metabolites. Compared to the NC group, RT altered 183 metabolites, with 112 upregulated and 71 downregulated. Information on these differential metabolites is provided in Supplementary Table 1. KEGG analysis showed that the differential metabolites between RT and NC were primarily enriched in linoleic acid metabolism, ascorbic acid and aldehyde metabolism, arachidonic acid metabolism, arginine and proline metabolism, sulfur metabolism, niacin and nicotinamide metabolism, histidine metabolism, tryptophan metabolism, and cysteine and methionine metabolism (Figure 5E). We then identified metabolites that were reversed in the M-FMT group, finding that 40 differential metabolites were restored by M-FMT. These metabolites, which should be a focal point of further study, are listed in Supplementary Table 2. KEGG pathway analysis suggested that FMT may influence rat metabolism through pathways such as niacin and nicotinamide metabolism, starch and sucrose metabolism, the pentose phosphate pathway, tyrosine metabolism, and arginine and proline metabolism (Figure 5F).

### Correlation of gut microbiota and fecal metabolites

**Figure 6.**
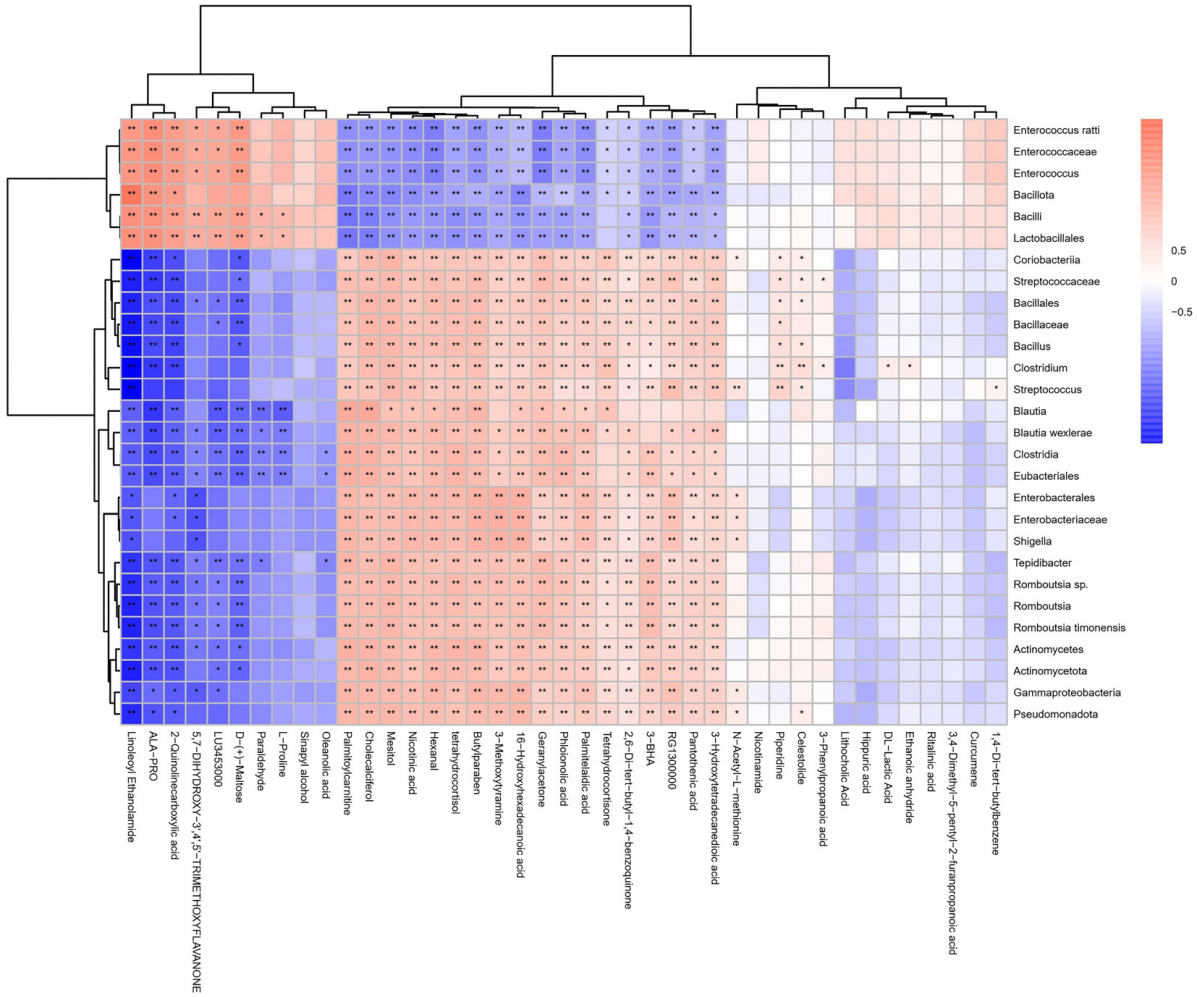
**The heatmap presents the Spearman correlation coefficients between fecal metabolites and significantly altered bacteria.** The intensity of the colors indicated the association degree (red, positive correlation; blue, negative correlation).

To clarify the potential relationship between gut microbiota and metabolites altered after FMT intervention, we conducted Spearman correlation coefficient analyses to examine associations between metabolites and microbial profiles (Figure 6). At the genus level, *Blautia*, *Romboutsia*, and *Clostridium*—which were restored following FMT—showed positive correlations with 18 metabolites, including 2,6-di-tert-butyl-1,4-benzoquinone, nicotinic acid, pantothenic acid, and palmitelaidic acid. Conversely, these genera were negatively correlated with ten metabolites, such as L-proline, propionaldehyde, D-(+)-maltose, linoleoyl ethanolamide, ALA-PRO, and 2-quinolinecarboxylic acid. In contrast, Enterococcus exhibited an inverse relationship with these metabolites. Similar patterns were observed at the species level, particularly with *Blautia wexlerae*, *Romboutsia timonensis*, *Romboutsia sp*., and *Enterococcus ratti*.

## Discussion

Radiotherapy is a key treatment modality for abdominal tumors, but a significant side effect is RE. In this study, we identified 12 Gy as the optimal dose for inducing an RE model. Additionally, our findings indicate that FMT can effectively alleviate RE symptoms by altering gut microbiota composition and associated metabolites. Further analysis suggests that these effects may be mediated through modulation of microbial profiles, particularly *Blautia*, *Romboutsia*, and *Clostridium*, which in turn may regulate pantothenic acid levels and influence niacin and nicotinamide metabolism. Clinically, nearly all patients undergoing pelvic or abdominal radiotherapy experience some form of GI symptoms, significantly diminishing their quality of life and imposing a substantial economic burden [33–35]. A key medical strategy for mitigating radiation-induced damage is a comprehensive understanding of radiation-related diseases. Research into the pathological mechanisms, prevention, treatment, and mitigation of radiation injury requires reliable animal models. Currently, RE models are primarily based on mice, rats, dogs, and rabbits. For instance, Shim et al. [36] used a single dose of 13 Gy to irradiate mice and demonstrated that rebamipide mitigated radiation-induced intestinal barrier damage by promoting intestinal cell proliferation and reducing inflammation. Li et al. [37] investigated the effects of umbilical cord mesenchymal stem cells (UCMSCs) on ARE in rats exposed to 10 Gy, while Hussein et al. [38] established a rat model irradiated with 8 Gy to explore the effects of melatonin on ARE induced by X-ray exposure. Given the lack of consensus on the optimal radiation dose for constructing an RE model, we systematically evaluated the most suitable dose for inducing RE in rats. In our study, SPF-grade SD rats were randomly assigned to receive different doses (10–16 Gy). Lower doses (10 Gy) caused insufficient damage, while higher doses (14 and 16 Gy) led to excessive damage and increased mortality. The 12-Gy dose effectively induced typical intestinal damage and inflammatory responses while maintaining a high survival rate, striking a balance between damage severity and physiological tolerance. This dose was suitable for subsequent studies and therapeutic evaluations. Notably, our results align with those reported by Li et al. [39].

The gastrointestinal tract harbors the most abundant microbial flora, and interactions between bacteria and receptors in local host tissues form a complex network that helps maintain physiological homeostasis. Gut microbiota plays a crucial role in the onset and progression of various diseases, including tumors and intestinal inflammation [40–42]. Recent studies have shown that radiotherapy disrupts the balance of intestinal microbiota in mice, significantly reducing microbial diversity, altering gut flora composition, and decreasing the survival rate of irradiated mice [43, 44]. Consequently, addressing radiotherapy-induced gut dysbiosis has become a key focus in the treatment of RE. FMT, which involves transferring intestinal flora from healthy donors to patients to restore gut microbiota, has emerged as a promising therapeutic approach [45, 46]. In pediatric inflammatory bowel disease, patients undergoing FMT exhibited reduced biodiversity and significant alterations in gut microbiota composition, characterized by an increase in *Enterobacteriaceae*, *Enterococcus*, *Haemophilus*, and *Fusobacterium* compared to donors, with a notable increase in species diversity 30 days post-FMT [20]. Additionally, in the context of radiation-induced toxicity, Cui et al. [47] demonstrated that FMT treatment increased the relative abundance of certain microbial populations, including *Bacteroidetes* (or *Lactobacillus*) and *Prevotella* at the genus level. In our study, rather than focusing on microbial changes at the genus level, as in previous research, we refined our analysis to the species level. Compared to the NC, *Enterococcus ratti* was significantly increased in the RT group, while *Blautia wexlerae*, *Romboutsia timonensis*, and *Romboutsia sp*. were significantly decreased. Following M-FMT, *Enterococcus ratti* was markedly reduced, whereas *Romboutsia timonensis* and *Romboutsia sp.* significantly increased. These findings suggest that FMT may alleviate RE symptoms by modulating the abundance of these specific microbiota. Beyond altering gut microbiota, radiation also induces significant changes in metabolite profiles. Recent studies suggest that intestinal flora contributes to radiation protection mechanisms through its co-metabolites [43, 48, 49]. Xiao et al. [50] found that FMT increased levels of the microbial-derived metabolite indole-3-propionic acid (IPA) in the feces of irradiated mice, reducing systemic inflammation, alleviating hematopoietic organ damage and myelosuppression, and improving gastrointestinal function and epithelial integrity. Similarly, Li et al. [51] demonstrated that FMT elevated levels of SCFAs in feces, while vitamin A supplementation improved the survival rate of irradiated mice, protected hematopoietic organs, and reduced gastrointestinal injury. To comprehensively analyze metabolite changes and identify potential biomarkers and metabolic pathways, we employed untargeted metabolomics analysis. Unlike targeted approaches, untargeted metabolomics captures unknown metabolites, allowing for a more holistic exploration of metabolic profiles. In our study, compared to the NC group, RT altered 184 metabolites, with 112 upregulated and 72 downregulated. We then identified 40 metabolites restored by FMT, which were enriched in pathways including niacin and nicotinamide metabolism, starch and sucrose metabolism, the pentose phosphate pathway, tyrosine metabolism, and arginine and proline metabolism. In the niacin and nicotinamide metabolism pathway, pantothenic acid was significantly decreased following irradiation but was upregulated after FMT. Notably, pantothenic acid showed positive correlations with microbiota, such as *Blautia*, *Romboutsia*, and *Clostridium*, which were significantly altered before and after treatment. Zhao et al. recently reported that pantothenic acid may enhance antioxidant status by modulating the JNK/p38 MAPK pathway and scavenging free radicals [52, 53]. Additionally, recent advancements in microbial fermentation, as demonstrated by Zou et al. [54], suggest that increasing pantothenic acid production could help alleviate inflammatory diseases.

In addition to the previously mentioned pathways, we observed elevated levels of fatty acid metabolites following FMT treatment, including palmitoylcarnitine, 3-hydroxytetradecanedioic acid, palmitelaidic acid, and 9,10-epoxyoctadecanoic acid. Correlation analysis revealed that *Romboutsia*, which decreased after radiotherapy, was positively associated with changes in these fatty acids. Prior studies suggest that *Romboutsia* plays a role in reducing inflammation and maintaining intestinal wall integrity [55]. Similarly, Li et al. [56] found that Jiawei Gegen Qinlian Decoction (PBM) not only increased *Romboutsia* levels but also enhanced propionate and total SCFA production in colitis rats. In patients with RE, SCFA production is often reduced due to intestinal inflammation and a low-fiber diet. Given that SCFAs are crucial for gut health, supplementation—either through dietary interventions or direct administration—may serve as a potential therapeutic strategy for inflammatory bowel disease. This is further supported by the use of SCFA enemas to alleviate RE symptoms [57]. Beyond medication and dietary interventions, daily care is equally vital in managing RE. Proper nursing care not only relieves symptoms and improves quality of life but also reduces the risk of recurrence. By monitoring symptoms, adjusting treatment plans, and providing psychological support, nursing staff help patients cope with stress and improve self-management. Additionally, personalized health education enhances patients’ understanding of their condition, treatment, and lifestyle modifications, ultimately improving medication adherence and fostering healthier habits. While FMT effectively restores gut microbiota balance and regulates metabolites—offering promise in treating recalcitrant diseases and reducing antibiotic dependence—our study has some limitations. The complexity of the procedure, challenges in donor screening, variability in daily care, and safety concerns remain significant hurdles. Moreover, our microbiological analysis could only determine the relative abundance of intestinal flora, not its precise composition. Additionally, the absence of established 16S rRNA references for rodent microbiomes limits interpretability. Finally, further research is needed to elucidate the precise mechanisms through which FMT influences intestinal microflora.

## Conclusion

Taken together, our data suggest that FMT effectively mitigates RE. The underlying mechanism appears to involve the regulation of intestinal flora and metabolites, with pantothenic acid and fatty acid supplementation emerging as potential therapeutic strategies for improving RE. However, further studies are needed to clarify the specific mechanisms behind these interactions.

## Supplemental data

Supplemental data are available at the following link: https://bjbms.org/ojs/index.php/bjbms/article/view/11835/3734.

## Data Availability

The original contributions presented in this study are included in the article/Supplementary Materials. Further inquiries can be directed to the corresponding author.
